# Effects of Combining Graphene Nanoplatelet and Phosphorous Flame Retardant as Additives on Mechanical Properties and Flame Retardancy of Epoxy Nanocomposite

**DOI:** 10.3390/polym12102349

**Published:** 2020-10-14

**Authors:** Woranan Netkueakul, Beatrice Fischer, Christian Walder, Frank Nüesch, Marcel Rees, Milijana Jovic, Sabyasachi Gaan, Peter Jacob, Jing Wang

**Affiliations:** 1 Institute of Environmental Engineering, ETH Zurich (Swiss Federal Institute of Technology Zurich), 8093 Zurich, Switzerland; woranan.netkueakul@empa.ch; 2 Laboratory for Advanced Analytical Technologies, Empa—Swiss Federal Laboratories for Materials Science and Technology, 8600 Dübendorf, Switzerland; 3 Laboratory for Functional Polymers, Empa—Swiss Federal Laboratories for Materials Science and Technology, 8600 Dübendorf, Switzerland; Beatrice.Fischer@empa.ch (B.F.); Christian.Walder@empa.ch (C.W.); Frank.Nueesch@empa.ch (F.N.); 4 Laboratory for Mechanical Systems Engineering, Empa—Swiss Federal Laboratories for Materials Science and Technology, 8600 Dübendorf, Switzerland; Marcel.Rees@empa.ch; 5 Additives and Chemistry Group, Advanced Fibers, Empa—Swiss Federal Laboratories for Materials Science and Technology, 9014 St. Gallen, Switzerland; milijana.jovic@empa.ch (M.J.); Sabyasachi.Gaan@empa.ch (S.G.); 6 Electronics and Reliability Center, Empa—Swiss Federal Laboratories for Materials Science and Technology, 8600 Dübendorf, Switzerland; Peter.Jacob@empa.ch

**Keywords:** nanocomposites, graphene nanoplatelets, mechanical properties, flame retardancy

## Abstract

The effects of combining 0.1–5 wt % graphene nanoplatelet (GNP) and 3–30 wt % phosphorous flame retardant, 9,10- dihydro-9-oxa-10-phosphaphenanthrene-10-oxide (DOPO) as fillers in epoxy polymer on the mechanical, flame retardancy, and electrical properties of the epoxy nanocomposites was investigated. GNP was homogeneously dispersed into the epoxy matrix using a solvent-free three-roll milling process, while DOPO was incorporated into the epoxy resin by mechanical stirring at elevated temperature. The incorporation of DOPO reduced the crosslinking density of the epoxy resin. When using polyetheramine as a hardener, the structural rigidity effect of DOPO overshadowed the crosslinking effect and governed the flexural moduli of epoxy/DOPO resins. The flexural moduli of the nanocomposites were improved by adding GNP up to 5 wt % and DOPO up to 30 wt %, whereas the flexural strengths deteriorated when the GNP and DOPO loading were higher than 1 wt % and 10 wt %, respectively. Limited by the adverse effects on mechanical property, the loading combinations of GNP and DOPO within the range of 0–1 wt % and 0–10 wt %, respectively, in epoxy resin were further studied. Flame retardancy index (FRI), which depended on three parameters obtained from cone calorimetry, was considered to evaluate the flame retardancy of the epoxy composites. DOPO showed better performance than GNP as the flame retardant additive, while combining DOPO and GNP could further improve FRI to some extent. With the combination of 0.5 wt % GNP and 10 wt % DOPO, improvement in both mechanical properties and flame retardant efficiency of the nanocomposite was observed. Such a combination did not affect the electrical conductivity of the nanocomposites since the percolation threshold was at 1.6 wt % GNP. Our results enhance the understanding of the structure–property relationship of additive-filled epoxy resin composites and serve as a property constraining guidance for the composite manufacturing.

## 1. Introduction

Epoxy resin (denoted as EP) is a thermoset polymer that has been extensively used for composite manufacturing due to its chemical and thermal resistance, excellent adhesion, and ease of processing [1]. Despite its attractive properties, the main disadvantages of epoxy resin are flammability [2], poor thermal conductivity, and brittleness. To manufacture epoxy thermosets, hardeners or curing agents can be employed for curing epoxy resin by crosslinking epoxy molecules forming the three-dimensional network. Among numerous kinds of hardeners such as amines, anhydrides, phenols, and thiols, amines have been the most widely used hardeners. The chemical structure of amines, i.e., aliphatic, cycloaliphatic and aromatic play important roles in the mechanical properties and thermal properties of the cured epoxy resin [3,4]. For example, due to the more rigid structure of 4,4’-diamino diphenyl methane (DDM), which is an aromatic amine, the epoxy thermoset cured with DDM showed higher glass transition temperature as compared to the epoxy thermoset cured with polyetheramine, which is an aliphatic amine [3]. Moreover, the type of hardener was chosen according to the desired properties and the applications of the resulting thermoset. For example, polyetheramine, an aliphatic amine, has been used for surface coatings, adhesives and castings for artistic purposes since aliphatic amines usually are colorless after curing. Aromatic amines such as 4,4’-diaminodiphenyl sulfone (DDS) provide excellent heat and chemical resistance, so it has been applied in the aerospace industry.

Various flame retardants and reinforcing nanomaterials have been investigated as epoxy resin fillers to overcome the flammability of epoxy thermosets. 9,10- dihydro-9-oxa-10-phosphaphenanthrene-10-oxide (DOPO) is one of the phosphorous flame retardants (PFRs), which were proposed as an alternative to halogenated flame retardants due to concerns about the latter’s toxic effects and environmental impacts [5,6]. Owing to its promising flame retardant efficiency, a number of studies have reported the effect of DOPO and its derivatives to enhance flame retardancy of epoxy composites [7,8,9,10,11]. The DOPO-incorporated epoxy resin cured with DDS and 2,4,6-tri (phenol-methylene-amide)-triazine with a phosphorous content of 3 wt % showed enhanced thermal stability as compared to the epoxy resin, but the glass transition temperature, flexural strength, and impact strength were decreased with increasing phosphorous content [7]. The flame inhibition mechanism of DOPO was proposed through a gas-phase reaction involving phosphorous oxide radical, PO, and H–PO fragment as reactive gas species [12]. However, incorporation of DOPO into epoxy resin is still a critical task because DOPO reacts with the epoxide group and thus can deteriorate the crosslinking density of epoxy resin, which is strongly dependent on the amount of epoxide groups, and might adversely affect the mechanical properties of the composites [7,10,13].

Graphene nanoplatelets (GNP) have been investigated as a filler in epoxy resin to enhance mechanical properties, thermal conductivity, electrical conductivity, and flame retardancy. Improved mechanical properties when adding GNP to epoxy resin have been reported, such as an increase in fracture toughness, flexural modulus, and hardness of the nanocomposites [14,15,16,17,18]. Wang and colleagues found a significant improvement in electrical conductivity of the epoxy/GNP nanocomposite by six orders of magnitude with 3 wt % GNP as compared to neat epoxy resin [19]. Previous studies revealed that GNP can delay the combustion process by retarding the formation of volatile gases and forming continuous and compact char that can block the radiation and decrease the combustion temperature, which can lead to an enhanced flame retardant effectiveness of polymer/GNP nanocomposites [20,21,22]. However, using GNP as a flame retardant is challenging due to poor dispersion of GNP in the polymer matrix, weak interaction between graphene surface and polymer, and strong tendency to agglomerate at high concentrations [23].

The combination of GNP with other flame retardants has gained attention to further enhance the properties of the nanocomposites in terms of mechanical performance as well as flame retarding efficiency. Recent studies reported that the combination of DOPO and GNP could improve flame retardancy of epoxy thermosets [24,25]. For example, the combination of DOPO and GNP showed a synergistic effect on flame retardancy properties of epoxy nanocomposites, i.e., by adding 2.5 wt % DOPO and 2.5 wt % GNP, the peak heat release rate was significantly reduced from 1194 to 396 kW/m^2^ [24]. Although studies have focused on the effects of combining GNP and DOPO on the flame retardancy property, there has not yet been an elaborate investigation on the effect of combining GNP and DOPO on mechanical properties of the composite. Especially, the role of DOPO in mechanical properties of the epoxy composites is still unclear. The incorporation of DOPO could cause negative effects on the mechanical properties of epoxy resin due to the decrease in crosslinking density by the reaction between DOPO and epoxy resin, which consumes epoxide groups [7,13]. However, some studies demonstrated that the incorporation of DOPO did not deteriorate the mechanical properties of the epoxy resin [26,27]. Wang and colleagues suggested that the mechanical properties of the epoxy/DOPO composites was not adversely affected because the constraining effect of the bulky DOPO group for the epoxy chain rotation could counteract the effect of the loss in crosslinking density [26]. In addition, too high concentration of GNP can lead to GNP agglomeration and worsen mechanical properties [23]. To our best knowledge, the effect of incorporation of DOPO as well as the combination of DOPO and GNP on the mechanical properties of the epoxy resin is still ambiguous and needs to be further studied.

Based on structural classification, the above mentioned studies [7,26,27] employed the aromatic hardeners resulting in deteriorating mechanical properties when the DOPO amounts exceeded certain thresholds. In our study, we aim to study the influence of DOPO on the mechanical properties of epoxy nanocomposite with another type of hardener as compared to previous studies. Therefore, we used an aliphatic amine, polyetheramine Jeffamine D-230, as a curing agent for epoxy resin (diglycidyl ether of bisphenol A). The solvent-free three-roll milling process was employed to disperse the GNP in the epoxy resin matrix. The influence of DOPO on the three-dimensional network structure of the epoxy resin was studied by assessing the crosslinking density. The flexural moduli of the epoxy nanocomposites with different loadings of DOPO and GNP were determined. Based on the mechanical properties, the maximum loading amounts of DOPO and GNP and their combination were chosen for further investigations. Flame retardancy of the composites was studied using cone calorimetry and the flame retardancy index (FRI) was applied to compare the flame retardancy efficiency among different samples. The results from this study revealed the appropriate formulation of DOPO-modified epoxy resin filled with GNP to achieve better overall performance especially the mechanical properties and flame retardancy. This study provides better understanding of the relationship between the structure of epoxy resin composites filled with two types of additives (DOPO and GNP) and their properties and facilitates the design of epoxy resin composites with desired properties.

## 2. Material and Methods

### 2.1. Materials

The epoxy resin used in this study was diglycidyl ether of bisphenol A (DGEBA, Araldite GY 250, Huntsman, Montgomery and Harris, TX, USA), which has an epoxy equivalent of 183–189 g·eq^−1^. The curing agent was polyetheramine Jeffamine D-230 (Huntsman, Montgomery and Harris, TX, USA), which has molecular weight (MW) of 230 g·mol^−1^ and amine hydrogen equivalent weight (AHEW) of 60 g·eq^−1^. The phosphorous-containing flame retardant 9,10- dihydro-9-oxa-10-phosphaphenanthrene-10-oxide (DOPO, MW = 216 g·mol^−1^) was purchased from Tokyo Chemical Industry (Tokyo, Japan). Graphene nanoplatelet xGNP M25 (carbon content > 99.5%, average particle diameters = 25 µm, thickness = 6–8 nm, typical surface area = 120–150 m^2^/g and density = 2.2 g·cm^−3^) was purchased from XG Sciences, St. Louis, MO, USA.

### 2.2. Incorporation of DOPO into Epoxy Resin

The desired amount of DOPO was added into the epoxy resin. The mixture was then heated with continuous stirring until the temperature reached 160 °C and kept constant with continuous stirring for 5 h for complete reaction between DOPO and epoxy resin [26]. The amount of DOPO was 3 wt %, 10 wt %, 20 wt % and 30 wt % of the epoxy resin matrix, which corresponded to the phosphorous content of 0.3, 1.0, 2.0 and 2.9 wt %, respectively.

### 2.3. Dispersion of GNP in Epoxy Resin

The dispersion method and manufacturing process of the composites was adapted from a previous study [28]. The desired amounts of GNP were added to epoxy resin or DOPO-incorporated epoxy resin (EP/DOPO). The components were mechanically mixed at 2000 rpm using an overhead mixer for 5 min. A three-roll mill machine (SDY200, Bühler AG, Uzwil, Switzerland) was employed to improve the dispersion of GNP in the polymer matrix. The mixture was passed through the three-roll mill for at least three times. Neat epoxy polymer was also prepared with the same method without any fillers.

### 2.4. Processing of Epoxy Resin

The ratio of resin/hardener was 100:32 for the composites without DOPO. For the EP/DOPO mixtures, since DOPO reacted with the epoxy groups, the amount of hardener added was adjusted according to the available epoxy groups obtained from the theoretical value as shown in the supplementary information. The mixture was mixed at 2000 rpm for 5 min. Afterward, the mixture was degassed at 80 °C for 2 min under vacuum. The formulation was transferred to a metal mold and cured at 80 °C for 12 h, and post-cured at 120 °C for 4 h. The mold was preheated at 80 °C for samples containing DOPO. All samples were allowed to cool down slowly to room temperature.

### 2.5. Characterization of GNP and EP/GNP/DOPO Composites and Measurement Procedures

Attenuated total reflection-Fourier transform infrared spectrometer (Agilent 640 FTIR spectrometer, Agilent technologies, Santa Clara, CA, USA) was used to characterize the functional groups of DOPO, epoxy resin, cured epoxy resins and cured EP/DOPO. The dispersion state of GNPs in the epoxy matrix was determined using an optical microscope (Zeiss, Oberkochen, Germany).

A discharge test was performed using an electrostatic Wimshurst machine as a high voltage source and the samples were placed adjacent to the spark gap formed by two metal spheres. The surface resistance was measured using a Keithley multimeter (model DMM7510, Keithley Instruments, Cleveland, OH, USA) with two-point electrodes. The samples had dimensions of 10 cm × 10 cm and 4 mm thickness.

The flexural modulus was evaluated by the three-point bending test according to ISO 178:2001 [29] using the Zwick Roell Z010 testing machine (ZwickRoell, Ulm, Germany) with a constant loading speed of 1 mm·min^−1^. Five specimens from each sample were tested. The dimensions of the specimen were 1 cm × 8 cm × 4 cm (width × length × thickness). Scanning electron microscope (Nova NanoSEM 230, FEI company, Hillsboro, OR, USA) was employed to analyze the fracture surface of the samples after a three-point bending test.

In order to determine the crosslinking density, dynamic mechanical thermal analysis (DMTA) was performed using an advanced rheometric expansion system (Rheometric Scientific, Piscataway, NJ, USA) to obtain the storage elastic modulus and tan δ of the cured resins, which were important parameters for an estimation of the crosslinking density. The samples tested were neat epoxy and EP/10DOPO. Samples were analyzed with applying constant static force at 1 Hz and a strain of 0.05%. The scanning temperature was from −150 to 180 °C at a heating rate of 3 °C·min^−1^. The sample dimensions were 10 mm × 50 mm and 4 mm thickness. The crosslinking density $\left(\rho \left(E^{\prime }\right)\right)$ in mol·m^−3^ was evaluated using the kinetic theory of rubber elasticity as follows:

(1) $$\rho \left(E\prime \right)=\frac{E^{\prime }}{3RT},$$

where *E’* is storage elastic modulus (Pa) of cured resin at the peak temperature of tan δ + 40 °C to ensure the rubbery stage of the sample. *R* is the gas constant (8.3145 m^3^·Pa·mol^−1^·K^−1^) and *T* is the absolute temperature (K) at which E’ is determined, in this case at the peak temperature of tan δ + 40 °C.

The glass transition temperature (T_g_) was analyzed using a differential scanning calorimeter (DSC 8000, Perkin Elmer, Waltham, Massachusetts, USA) with a heating rate of 20 °C·min^−1^. The scanned temperature ranged from 20 to 200 °C. Thermal stability was measured by a thermogravimetric analysis using Thermobalance Netzsch TG209 F1 (NETZSCH-Gerätebau GmbH, Selb, Germany) under a nitrogen environment at the heating rate of 20 °C·min^−1^ from 28 to 1008 °C. Several parameters were obtained to describe the thermal stability of cured epoxy resins. The onset temperature or initial decomposition temperature (T_d_) is the temperature at which the sample starts to decompose. The mid-point temperature (T_−50%_) is the temperature at which 50% of the weight loss occurred. T_max_ stands for the decomposition temperature at the maximum mass loss rate. The amount of final residue presented by % char is the percentage of final residual weight at 1008 °C with relative to the initial sample weight.

The FTT Cone calorimeter (Fire Testing Technology, West Sussex, UK) was employed to analyze the flammability of the composites according to ISO 5660-1 with the heat flux of 50 kW·m^−2^. The experiments were terminated after the flame stopped for 100 s. The sample size was 10 cm × 10 cm × 4 mm (length × width × thickness).

## 3. Results and Discussion

### 3.1. Verification of the Incorporation of DOPO into Epoxy Resin using ATR-FTIR

The successful incorporation of DOPO into the epoxy resin, whose reaction is presented in Figure S1, was confirmed by FTIR spectroscopy (Figure S2). The P–H stretching vibration peak of DOPO appeared at 2384 cm^−1^. The disappearance of this peak confirmed the bonding of DGEBA and DOPO [10]. The reaction between DGEBA and DOPO led to the epoxide ring opening that resulted in the formation of hydroxyl group on the sp^3^ carbon [26]. The formed hydroxyl group could further react with another epoxide ring via the etherification reaction, which subsequently formed the C–O–C (alkoxy) bond and a hydroxyl group (O–H) [26]. The occurrence of this reaction could be evidenced in the EP/DOPO sample from the presence of the alkoxy C–O bond at 1117 cm^−1^ and the existence of hydroxyl groups as a broad peak between 3200 and 3600 cm^−1^, which was broader and more intense as compared to the O–H peak of the neat epoxy resin, which appeared between 3400 and 3600 cm^−1^. Moreover, the shift of the P–C stretching vibration from 682 cm^−1^ in DOPO to 686 cm^−1^ in EP/DOPO could also be a sign of the bonding between the phosphorus atom of DOPO and a carbon atom of epoxide ring.

### 3.2. Dispersion of GNP in the Epoxy Resin

**Figure 1:**
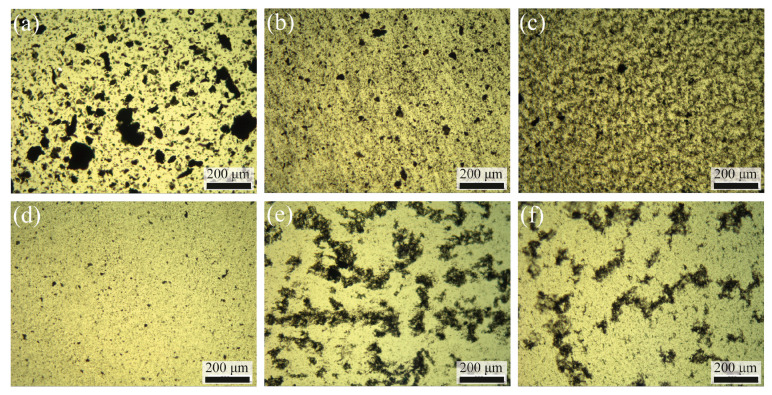
Light micrographs illustrating the dispersion degree of GNP in the epoxy resin (**a**) after mixing the epoxy resin with 0.5 wt % GNP using a high speed mixer, (**b**) and (**c**) after one and three runs of three-roll milling, (**d**) after adding the hardener, and (**e**) and (**f**) after curing at room temperature and at 80 °C, respectively.

Figure 1 shows the dispersion state of GNP in the epoxy resin matrix. Heterogeneity in lateral size and dispersion state of GNPs was observed after mixing GNP and epoxy resin with a high-speed mixer for 5 min. The lateral dimension of GNPs substantially reduced and the dispersion of GNPs was more homogeneous after one and three runs of three-roll milling. After adding the hardener, the GNP concentration decreased due to the dilution by the added hardener. Reagglomeration of GNPs was observed for the samples cured at both room temperature and 80 °C. The reagglomeration during the curing process was expected and previously reported for reduced graphene oxide [30] and carbon nanotube [31,32]. Since the curing rate is faster at higher temperature, the diffusion of GNP could be more limited by a sharp increase in viscosity of the resin during the curing process at higher temperature [31]. Therefore, we assumed that curing the epoxy resin at 80 °C yielded better GNP dispersion.

### 3.3. Electrical Property

The discharge measurement (Table S3) revealed that the nanocomposites with GNP content higher than 1 wt % showed an electrical discharge from the metal spheres to the samples, implying that the nanocomposites contained conductive networks. Corona discharge appeared for the nanocomposites with 2–3 wt % GNP, indicating the suitability for antistatic packaging purpose [33]. The nanocomposites containing less than 1 wt % GNP were completely insulating as the line spark appeared between two metal spheres.

**Figure 2:**
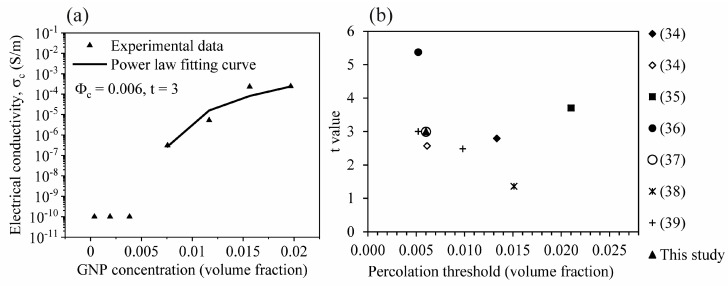
(**a**) Percolation threshold of the EP/GNP nanocomposites in this study and (**b**) comparison of percolation threshold and t values among EP/GNP nanocomposites from different studies.

Percolation theory can be applied to predict the critical amount of the filler that can form the connected network and affect the behaviors of the nanocomposite. The electrical percolation threshold, ϕ_c_, and critical exponent, t, obtained from fitting the experimental data with the power law $\sigma_{c}=\sigma_{f}{\left(\Phi -\Phi_{c}\right)}^{t}$ were 0.006 and 3, respectively as demonstrated in Figure 2a. This corresponds to a volume fraction ϕ_c_ of 0.006 or 1.6 wt % GNP. σ_c_ and σ_f_ are the conductivity of the nanocomposite and the filler, respectively, and ϕ is the filler concentration.

The percolation threshold values published earlier ranged from 0.0052 to 0.021 volume fraction, while the corresponding t values were in the range of 1.36–5.37 as summarized in Figure 2b [34,35,36,37,38,39]. Higher t value than the universal value (1.65–2) indicates the nonuniversal transport behavior, which can happen when the fillers have exceptional geometries such as a high aspect ratio like GNP [40]. An analytical model $\sigma_{c}=\frac{27\pi D^{2}t_{p}}{4{\left(D+IPD\right)}^{3}}$ was also proposed to predict the percolation threshold of nanocomposites containing three-dimensional randomly dispersed disc-shaped or platelet-like nanoparticles based on average interparticle distance (IPD) and aspect ratio of the particles, where D is the diameter of the platelet and t_p_ is the thickness of the platelet [41]. The suggested IPD value was 10 nm [41], at which the electron hopping can occur, according to the quantum mechanical tunneling mechanism. Regarding GNP with the diameter and thickness of 25 µm and 6–8 nm, respectively, the estimated percolation threshold was between 0.0051 and 0.0068 volume fraction, which is in good agreement with the calculated percolation threshold from the power law fitting of our experimental data.

### 3.4. Crosslinking Density of Epoxy Resins

Properties of the epoxy resins were influenced by the chemical network structure, which could be explained by the crosslinking state of the cured epoxy resins [42]. In order to assess the effect of DOPO on the network structure of the cured resin, the crosslinking density of EP/10DOPO was estimated (Equation (1)) and compared to that of neat epoxy using the rubbery storage modulus (E’), which was E’ at the peak temperature of tan δ + 40 °C, obtained from DMTA results (Figure S3). The peak temperature of tan δ + 40 °C was 136 °C. The results involved in the determination of the crosslinking density are presented in Table 1.

As shown in Table 1, the crosslinking density of the EP/10DOPO was lower than that of neat epoxy. This was a result of the reaction of epoxide groups with DOPO, causing the depletion of available epoxide groups and less crosslinking reaction between epoxide groups and amine groups of the hardener.

### 3.5. Mechanical Properties

**Figure 3:**
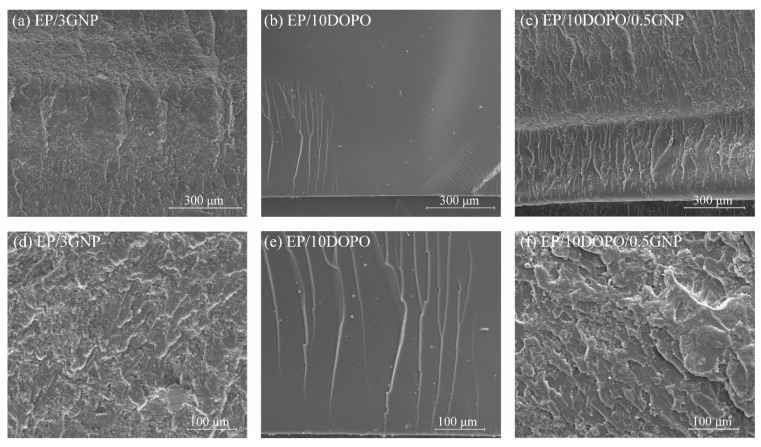
Scanning electron microscopy (SEM) images of fracture surface after three-point bending test of (**a**), (**d**) EP/3GNP, (**b**), (**e**) EP/10DOPO, and (**c**), (**f**) EP/10DOPO/0.5GNP.

The flexural fracture surface of the epoxy composites was determined using SEM as shown in Figure 3. The fracture surface of the formulation containing GNP showed a rough feature. The roughness on the EP/GNP nanocomposites was caused by GNP acting as an obstruction of the crack propagation, which resulted in an alteration of the crack path as seen in Figure 3a,c. Moreover, the rough surface included holes and GNP agglomerates on the fracture surface (Figure 3d,f) suggesting adhesive failure of the composite. The fracture surface of EP/10DOPO (Figure 3b,e) revealed mirror-like feature with some cracks, which was similar to that of neat epoxy resin.

**Figure 4:**
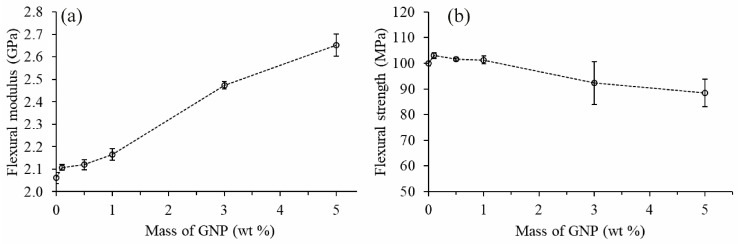
Effect of GNP on the (**a**) flexural modulus and (**b**) flexural strength of EP/GNP nanocomposites.

Flexural modulus and flexural strength were investigated to describe the mechanical performance of the EP/GNP nanocomposites as displayed in Figure 4. We found an increase in flexural modulus by 20% and 29% compared to neat epoxy resin when the amount of GNP increased to 3 wt % and 5 wt %, respectively. The flexural strength of the nanocomposite slightly increased as GNP content increased up to 1 wt %. When GNP content increased to 3 wt % and 5 wt %, a decreasing trend of flexural strength of the nanocomposites was observed due to the weak adherence between the surface of epoxy resin matrix and non-functionalized GNP used in this study, which prevent the load transfer from matrix to GNP. Therefore, GNP could act as a stress concentrator and deteriorate the mechanical properties of the nanocomposites [18,43]. This could be confirmed by the fracture surface analyzed by SEM (Figure 3) revealing rough surface with voids and GNP agglomerates, which indicated the adhesion failure between the matrix and GNP.

**Figure 5:**
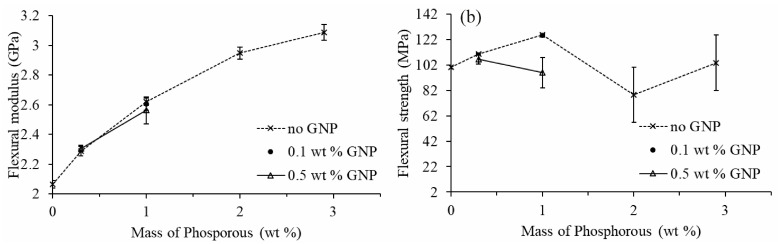
Effect of DOPO on the (**a**) flexural modulus and (**b**) flexural strength of DOPO-incorporated epoxy nanocomposites.

Figure 5 shows the effect of incorporating DOPO into epoxy resin on the flexural modulus and flexural strength. The addition of DOPO led to a significant increase in the flexural modulus. In the presence of DOPO, the addition of GNP did not significantly alter the flexural modulus and strength of the nanocomposite as compared to the EP/DOPO composites. The incorporation of DOPO up to 10 wt % (or 1 wt % of phosphorus) showed an improvement in flexural strength of the nanocomposites, whereas adding a higher amount of DOPO adversely affected the flexural strength. The mechanical effect of DOPO in the epoxy thermoset is still unclear due to a lack of data and the inconsistent results among different studies [7,13,26,27]. The incorporation of DOPO was reported to cause a negative impact on the flexural modulus and strength of the cured epoxy resins [7,13] because of the reaction of DOPO with the epoxide group, which reduced the functionality of the epoxy resin and thus diminished the degree of crosslinking in the resulting thermosets [26]. On the other hand, the rigidifying effect of DOPO once linked to the epoxy chain might compensate the crosslinking effect; therefore, the addition of DOPO to a certain concentration level led to an increase in the flexural modulus of the epoxy thermosets [26,27].

**Figure 6:**
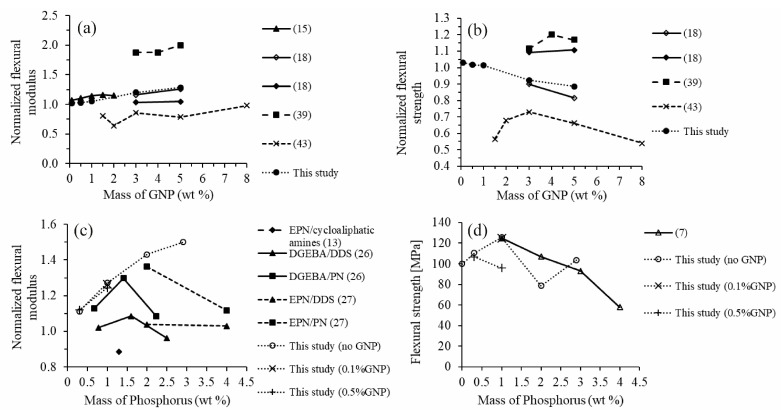
Comparison among different studies of the effect of GNP on the (**a**) flexural modulus and (**b**) flexural strength of epoxy nanocomposites normalized to neat epoxy resin and the effect of DOPO on the (**c**) flexural modulus normalized to neat epoxy resin and (**d**) flexural strength in MPa.

The flexural moduli and strengths of the nanocomposites measured in this study were compared with the values from other studies as shown in Figure 6 [15,18,39,43]. Since we are interested in the effects of DOPO and GNP incorporation, the flexural moduli and strengths were normalized to the cured neat epoxy resin so that the values from different studies could be compared. Regarding GNP addition, the improvement in flexural modulus could be mainly attributed to the good dispersion of GNP in the epoxy resin by three-roll milling as shown in Figure 1. Several previous studies also used three-roll milling [15,18,39,43,44] for GNP dispersion and showed improvement of the mechanical properties of the nanocomposites.

With an increasing amount of DOPO, our study showed a continuous improvement in the flexural modulus of the EP/DOPO thermosets, which does not completely agree with other studies, as shown in Figure 6c. Our study presents an increasing trend in the flexural modulus when phosphorous content in DOPO-incorporated epoxy resin rose up to 3 wt %. Other studies found a decrease in terms of the flexural modulus of the DOPO-incorporated epoxy resin as the phosphorous content increased to some extent. For example, Wang and Lin [26] reported an improvement in flexural modulus of the DOPO-incorporated resin (DGEBA) cured with 4,4′-diaminodiphenyl sulfone (DDS) and phenol novolac (PN) when the phosphorous content increased to 1.6 wt % and 1.4 wt %, respectively. When the phosphorous content exceeded these values, the flexural modulus decreased. Wang et al. [13] examined the flexural modulus of the DOPO-incorporated epoxy novolac resin (EPN) with 1.3 wt % phosphorous cured with modified cycloaliphatic amines. They found that the flexural modulus of the DOPO-modified epoxy resin was worsened by 11% as compared to neat resin, but it could be improved by adding 0.5 wt % of graphene into the formulation.

Such a discrepancy in the DOPO effect on the flexural modulus of the cured DOPO-modified epoxy resin could result from different types of hardeners used in various studies since the structure of the hardeners could also play an important role on the mechanical properties of the epoxy thermosets [3,45,46]. Hardeners containing aromatic rings such as phenol novolac (PN) and 4,4′-diaminodiphenyl sulfone (DDS) [26,27] or cycloaliphatic structure [13] could also enhance the flexural modulus or rigidity of the epoxy thermoset. Considering the molecular structure of the involved compounds, the rigidity of DOPO and aromatic hardeners could contribute to an improvement in flexural modulus of the cured DOPO-incorporated resin. When the load of DOPO increased, the available epoxide groups decreased due to the reaction of DOPO with some of the epoxide groups and thus less of an amount of hardener was required to crosslink DOPO-incorporated epoxy resin. Therefore, the flexural modulus could be strengthened by the rigidity of DOPO; meanwhile, it could be weakened by both the loss of crosslinking density and reduction in aromatic structures caused by less of an amount of hardener required. On the other hand, hardeners with a linear molecular structure did not contribute strongly to the mechanical properties of the epoxy thermoset; therefore, the rigidifying effect of DOPO was the dominant factor on the flexural modulus and the flexural modulus increased continuously when phosphorus content increased. The competition of the above strengthening and weakening effects was the reason for the different slopes of flexural modulus versus phosphorus content curves from previous studies [26,27] in Figure 6c.

### 3.6. Thermal Properties

**Figure 7:**
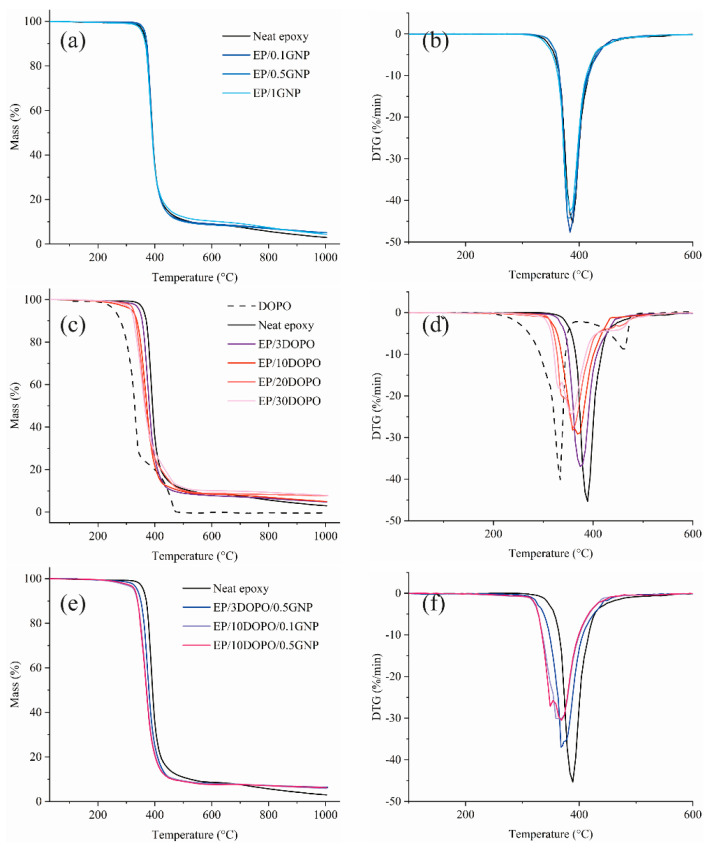
(**a**,**c**,**e**) TGA and (**b**,**d**,**f**) DTG curvesfrom thermogravimetric analysis under the nitrogen environment of DOPO, GNP, cured neat epoxy resin, EP/GNP, EP/DOPO, and EP/DOPO/GNP.

TGA curves and their derivatives (derivative thermogravimetric, DTG), which represent the thermal stability of the samples are demonstrated in Figure 7. EP/GNP samples had similar TGA profile as compared to neat epoxy resin, which showed T_d_ around 369 °C and the maximum mass loss rate around 387 °C. However, GNP significantly increased the char residues of the epoxy. The addition of GNP up to 1 wt % loading can increase the char content up to 50% by forming the structure containing smaller pores as compared to the char of neat epoxy, which led to a compact and continuous char structure [20]. DOPO underwent the mass loss in two steps from 200 to 370 °C and from 370 to 480 °C, respectively, which was earlier than the T_d_ of neat epoxy resin at 369 °C. After 480 °C, DOPO completely decomposed, as there was almost no residue left. Regarding EP/DOPO samples, when the DOPO loading increased, T_d_ and T_max_ of the EP/DOPO decreased. Our results clearly showed significant improvement in char yields as the amount of DOPO increased. This could be due to the fact that the decomposition products of DOPO could catalyze the formation of char, which can insulate the underneath layer from burning [13,24,47]. Adding GNP to the EP/DOPO composite did not affect T_d_ and T_max_ of the epoxy resin as compared to EP/DOPO, but it could further improve the char residues.

Table 2 summarizes the glass transition temperatures (T_g_) of epoxy nanocomposites determined by DSC and the important parameters obtained from thermal stability investigation by TGA. GNP slightly reduced T_g_ of the cured epoxy resin as compared to the neat epoxy resin. The reduction in T_g_ could be the result of GNP acting as a heat flow barrier in the polymer matrix [48]. Moreover, non-functionalized GNP had free surface and formed repulsive interface with the polymer, which could result in a decrease in T_g_ because polymer chain mobility was more enhanced with respect to the neat epoxy resin [49,50].

An increase in the DOPO content resulted in a decrease in T_g_ due to the reaction of epoxide groups and DOPO, which depleted the available crosslinking sites between epoxy resin and hardener and resulted in less crosslinking network of the cured DOPO-incorporated epoxy resin. The addition of GNP to EP/DOPO did not make a notable difference in T_g_ and T_d_ of the resulting nanocomposites as compared to EP/DOPO samples; however, the significant improvement in char yield was observed in samples containing GNP.

### 3.7. Flame Retardancy

**Figure 8:**
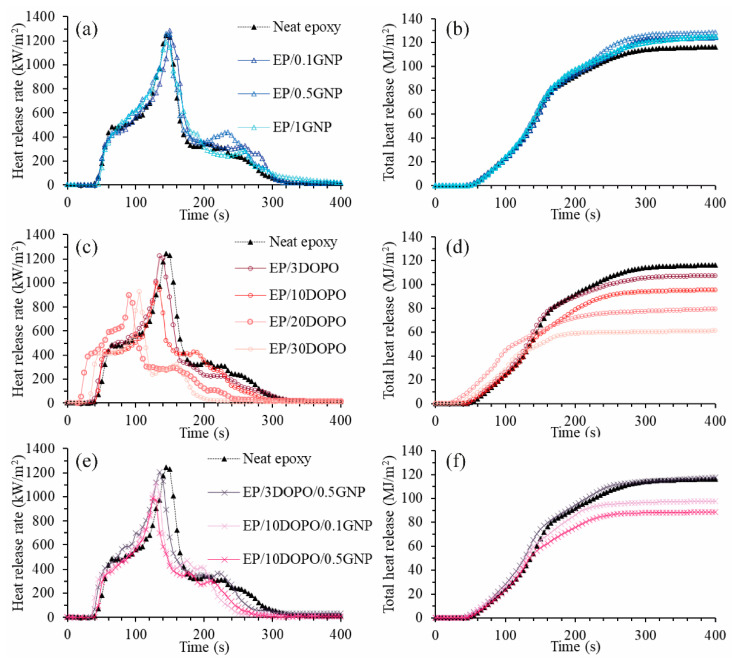
Heat release rate plots from cone calorimetry of neat epoxy resin compared to (**a**) EP/GNP nanocomposites, (**b**) EP/DOPO, and (**c**) EP/DOPO/GNP nanocomposites and total heat release plots from cone calorimetry of neat epoxy resin compared to (**d**) EP/GNP nanocomposites, (**e**) EP/DOPO, and (**f**) EP/DOPO/GNP nanocomposites.

The important parameters for flame retardancy including time to ignition (TTI), peak heat release rate (pHRR), average heat release rate (ave-HRR), average effective heat of combustion (ave-EHC), total heat release (THR), total smoke production (TSP), average CO formation (ave-CO yield), and average CO_2_ formation (ave-CO_2_ yield) were obtained from cone calorimetry. Figure 8 shows plots of HRR and THR as a function of burning time from cone calorimetry analysis. HRR profile of neat epoxy resin showed pHRR of 1246 kW·m^−^^2^ at 140 s. GNP did not significantly influence pHRR, but another HRR peak around 200–300 s was more pronounced as compared to that of the neat epoxy. The development of the second peak in the HRR profile of EP/GNP could be an effect of the breakage of the char layer formed by GNP. When the char layer broke, the combustible gases were released, which enhanced the combustion. As a result, THR of the EP/GNP was higher than that of neat epoxy. Liu et al. reported a similar effect of GNP on the development of small HRR peaks with the addition of 5 wt % of GNP; however, they showed substantial reduction of pHRR by 57% as compared to the epoxy resin without a filler [24], while our EP/GNP did not reveal an obvious reduction in pHRR. This might be explained by the fact that the concentration of GNP in our study was lower and thus the protective char layer formed was weaker and easier to break.

The incorporation of DOPO could decrease the pHRR and THR in relation to the epoxy resin because DOPO could act in both the condensed phase by forming the protective char layer and gas phase by scavenging the reactive combustible species [12]. By adding GNP into EP/DOPO, the pHRR and THR were further reduced as compared to EP/DOPO.

Table 3 summarizes some important parameters from cone calorimetry. Moreover, based on TTI, pHRR, and THR, the flame retardancy index (FRI), which is a dimensionless value defined as $FRI=\;\frac{{\left(THR\times \frac{pHRR}{TTI}\right)}_{neat\;epoxy}}{{\left(THR\times \frac{pHRR}{TTI}\right)}_{composite}}$ [51], was calculated in order to compare the effect of adding GNP, DOPO, and the combination of both additives on the flame retardancy efficiency of the epoxy resins. The addition of GNP could increase the TTI, which could be an effect of GNP changing the gas pathway in the condensed phase and retarding the gas transfer to the gas phase. The high thermal diffusivity of GNP could also contribute to the delay of TTI as GNP could transfer the heat from the surface of the composite to the bulk [52]. On the other hand, when the loading amounts of DOPO increased, the TTI did not show an increasing trend. This might be due to the relatively low T_d_ of DOPO as compared to the neat epoxy resin as shown in TGA results. TSP, ave-CO yield, and ave-CO_2_ yield are indicators of the completeness of the combustion. The products of complete combustion are water and CO_2_, while incomplete combustion produces smoke and CO [53]. Addition of DOPO resulted in higher TSP, greater development of CO, and lower CO_2_ production, which indicated more incomplete combustion as compared to the neat epoxy resin. The incorporation of DOPO reduced pHRR, ave-HRR, ave-EHC, and THR of the composites as compared to the cured neat epoxy resin. According to our finding, GNP up to 1 wt % did not significantly promote the flame retarding efficiency of the epoxy resin in terms of pHRR and THR. However, the combination of DOPO and GNP could further reduce pHRR, ave-HRR, and THR of the nanocomposites as compared to the EP/DOPO or EP/GNP alone.

The flame retarding mechanism of combining DOPO and GNP has been proposed as GNP is acting in the condensed phase, while DOPO is acting in both the condensed phase and gas phase [24,47]. Ave-EHC reflects the combustion rate of volatile compounds in the gas phase during combustion [24,47]. A significant decrease in ave-EHC was observed for EP/DOPO composites, implying that DOPO reacts in the gas phase by scavenging the volatile compounds and thus resulted in the reduction of the burning rate. On the other hand, GNP did not reduce the ave-EHC when added to the composite, which suggests that GNP did not contribute to the gas phase reaction.

The change in TTI, pHRR, and THR with respect to the neat epoxy resin was reflected in FRI values of the composites. Three performance categories were proposed including poor, good, and excellent, corresponding to FRI < 1, 1 < FRI < 10, and FRI > 10, respectively [51]. The results revealed that adding GNP slightly improved FRI due to the effect of GNP on delaying the TTI of the EP/GNP nanocomposites, although it caused an increase in pHRR and THR. In case of DOPO, when DOPO loading increased, the pHRR and THR reduced, which resulted in an increase in FRI, even though the TTI was not delayed. It can be seen that all samples except for EP/0.5GNP, EP/3DOPO, and EP/3DOPO/0.5GNP showed good flame retardancy performance, whereas EP/0.5GNP, EP/3DOPO, and EP/3DOPO/0.5GNP showed poor FRI quantities. Regarding EP/0.5GNP, as mentioned earlier, the formed char layer was not strong enough, resulting in the breakage of the char layer, which caused a release of combustible gases and an increase of THR. Moreover, pHRR was not decreased by adding GNP. These were major contributions to poor FRI. Since ave-EHC, CO, and CO_2_ production, which reflected the chemical reaction process [20], of EP/0.5DOPO were similar to those of the neat epoxy resin, this indicated that GNP did not interfere with the gas phase reaction. For EP/3DOPO, the poor FRI was mainly due to low TTI, which could be a result of relatively low T_d_ of DOPO as compared to the neat epoxy resin, meaning that EP/3DOPO decomposed earlier than neat epoxy resin, as evidenced in the TGA results. Combining 0.5 wt % GNP with 3 wt % DOPO could delay TTI of the epoxy composite, but THR was increased, which was a major contribution to the poor FRI. In both cases, avg-EHC did not decrease, indicating that no gas phase reaction occurred. Since DOPO was responsible for the effect on the gas phase reaction, we concluded that the loading of 3 wt % DOPO in our epoxy system was not enough for DOPO to affect the gas phase reaction. The combination of 10 wt % DOPO and 0.5 wt % GNP could additionally promote the FRI to 1.9 as compared to the epoxy resin loaded with 10 wt % DOPO, which showed FRI at 1.7.

Since FRI is a relative value indicating the flame retardancy efficiency of the composites with respect to the neat epoxy resin, this allows us to compare the influence of additives on the flame retardancy efficiency of different epoxy resin systems. Our results demonstrated that, in terms of the FRI values, DOPO showed better flame retardancy performance as compared to GNP. Our finding was consistent with the previous review work, which extracted the information regarding flame retardancy efficiency from a number of literature [54]. They reported that generally phosphorous-containing flame retardants were more effective in terms of flame retardancy in epoxy resin as compared to nanoparticle fillers [54]. Specifically, they showed that DOPO-incorporated epoxy resins with the DOPO loading ranging from 1.3 to 13 wt % had an FRI in the range of 0.8 to 3.94. Regarding graphene-related materials including GNP, expanded graphite, graphene oxide and reduced graphene oxide, with the nanoparticle loadings ranged from 1 to 30 wt %, the FRI of the epoxy nanocomposites were in the range of 0.98 to 2.34. It can be seen that epoxy filled with DOPO could achieve better FRI than epoxy filled with graphene-related materials, while loadings of DOPO (1.3–13 wt %) were less than loadings of graphene-related materials (1–30 wt %).

Studies have reported enhanced flame retardancy property by adding graphene-related materials. For example, when 5 wt % of multilayer graphene was added to polypropylene and 5 wt % of graphene nanosheets were added to epoxy resin, they could achieve FRI of 2.9 [21] and 4.4 [24], respectively, which were considered good performance in terms of FRI [51]. However, the amounts of graphene-related materials used in those studies were at least 5 wt %, which could adversely affect the mechanical properties of the epoxy composite according to our findings. By combining DOPO and GNP, the loading amount of GNP could be reduced and the flame retardancy property could be improved without worsening the mechanical properties of the nanocomposites, which was demonstrated in this work.

As shown in our study, the flame retardancy efficiency of epoxy resins could be enhanced by the incorporation of DOPO since DOPO could act in both the condensed phase and gas phase to retard the flame. By combining DOPO and GNP, the flame retardancy efficiency could be further improved to some extent as compared to the EP/DOPO composite due to the effect of GNP on delaying the ignition time and improving char formation. One might consider increasing the GNP loading to achieve better flame retardancy performance; however, a balance must be considered due to the adverse effect on the mechanical properties of the composites resulting from agglomeration of GNP at high loading.

## 4. Conclusions

Epoxy-based composites with homogeneously dispersed GNP were produced using a three-roll milling process. DOPO was also successfully incorporated into the epoxy resin. The electrical percolation threshold was detected at 0.006 volume fraction of GNP or 1.6 wt % GNP. Both DOPO and GNP could enhance the flexural modulus and flexural strength of the epoxy composites. Unlike aromatic hardeners, the linear structure of polyetheramine, which was the hardener used in this study, allowed the rigidity of grafted DOPO to dominate the flexural modulus of the composites; therefore, the flexural modulus increased continuously as the DOPO loading increased, although the crosslinking density decreased when DOPO was incorporated. However, higher DOPO and GNP contents (more than 10 wt % DOPO and 1 wt % GNP) could cause difficulty in composite manufacturing due to an increase in viscosity, which resulted in poorer dispersion of GNP and poorer flexural strength of the composites. Since incorporation of DOPO depleted the crosslinking density of the epoxy thermoset, the glass transition temperature decreased as DOPO loading increased. Thermogravimetric analysis showed that both GNP and DOPO could enhance the char residues. Incorporating 10 wt % DOPO could reduce the peak heat release rate (pHRR) and the total heat release (THR) of epoxy composites by 20% and 18%, respectively, compared to the neat epoxy resin. Increasing GNP loading up to 1 wt % did not significantly affect the pHRR of epoxy composites, but increased the THR by 8% and delayed TTI. These three parameters, pHRR, THR, and TTI could be converted to FRI. The results revealed that EP/DOPO had better FRI than EP/GNP. Using DOPO and GNP together could further improve FRI to some extent and achieve good performance regarding the FRI criterion.

**Figure 9:**
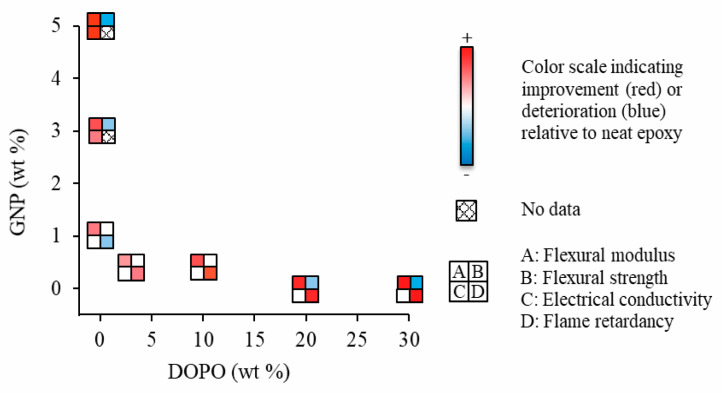
Comparison, with respect to neat epoxy resin, of the mechanical, electrical, and flame retarding properties of epoxy composites with different loading amounts of GNP and DOPO.

The effects of GNP and DOPO loading on the composites’ properties are illustrated in Figure 9. Here, based on a DGEBA/polyetheramine system, we proposed the combination of 10 wt % DOPO and 0.5 wt % GNP as an optimal formulation providing enhanced flexural modulus and strength, an improvement in flame retarding efficiency of the composite by lowering the pHRR and THR by 21% and 24%, respectively, and maintained electrical insulating properties of the epoxy resin.

## Figures and Tables

**Table 1:** Storage modulus (E’) in rubbery region and crosslinking density of neat epoxy resin and EP/10DOPO.

Samples	E’ at Peak Temperature of tan δ + 40 °C (MPa)	ρ (mol·m^−3^)
Neat epoxy	7.67	751.5
EP/10DOPO	6.36	623.2

**Table 2:** Glass transition temperatures (T_g_) and thermogravimetric analysis data of DOPO, neat epoxy, EP/GNP, EP/DOPO, and EP/DOPO/GNP nanocomposites.

Samples	T_g_ (°C)	T_d_ (°C)	T_−50%_ (°C)	T_max_ (°C)	% Char
DOPO	N.A.	279	329	333	0
Neat epoxy	98.0	369	389	387	2.88
EP/0.1GNP	91.8	370	389	386	5.07
EP/0.5GNP	95.1	368	389	381	5.06
EP/1GNP	95.8	366	389	385	4.31
EP/3DOPO	94.3	355	379	374	4.76
EP/10DOPO	87.4	339	369	370	4.92
EP/20DOPO	83.3	325	366	360	7.69
EP/30DOPO	72.8	319	363	357	7.80
EP/3DOPO/0.5GNP	94.1	353	378	369	6.32
EP/10DOPO/0.1GNP	88.1	340	369	362	5.81
EP/10DOPO/0.5GNP	84.9	338	369	368	6.14

**Table 3:** Parameters from cone calorimetry analysis including TTI, pHRR, ave-HRR, ave-EHC THR, TSP, ave-CO yield and ave-CO_2_ yield.

Samples	TTI (s)	pHRR (kW·m^−2^)	Ave-HRR (300s) (kW·m^−2^)	Ave-EHC (300 s) (MJ·kg)	THR (MJ·m^−^²)	TSP (m^2^)	Ave- CO yield (kg·kg^−1^)	Ave- CO_2_ yield (kg·kg^−1^)	FRI (-)
Neat epoxy	34	1246	384	21.3	117	30.3	0.05	1.6	-
EP/0.1GNP	40	1285	411	23.9	125	34.0	0.06	1.7	1.1
EP/0.5GNP	38	1263	425	24.8	130	33.7	0.06	1.8	1.0
EP/1GNP	42	1189	410	24.0	126	33.4	0.06	1.7	1.2
EP/3DOPO	28	1226	355	22.0	108	37.1	0.08	1.5	0.9
EP/10DOPO	37	996	315	19.5	96	36.4	0.11	1.3	1.7
EP/20DOPO	18	902	259	13.6	81	39.2	0.10	0.7	1.1
EP/30DOPO	29	927	201.1	13.9	60.1	35.7	0.10	0.8	2.3
EP/3DOPO/0.5GNP	34	1208	385.4	23.2	118	49.4	0.12	1.7	1.0
EP/10DOPO/0.1GNP	31	1116	323.7	20.9	98.4	38.5	0.12	1.3	1.2
EP/10DOPO/0.5GNP	38	989	294.2	19.1	89.3	35.5	0.11	1.3	1.9

## Supplementary Materials

The following are available online at https://www.mdpi.com/2073-4360/12/10/2349/s1, Figure S1: Reaction between epoxy resin (DGEBA) and DOPO, Figure S2: ATR-FTIR spectra indicating the functional groups of DOPO, neat epoxy and epoxy/DOPO mixture, Figure S3: Storage modulus (E’) and tan δ of neat epoxy resin and EP/10DOPO obtained from DMTA, Table S1: Formulations of epoxy resin and DOPO-incorporated epoxy resin cured with polyetheramine (Jeffamine D-230), Table S2: Identification of observed peaks from FTIR spectra of DOPO, neat epoxy and epoxy/DOPO mixtures, Table S3: Electrical properties of the epoxy/GNP composites.
