# Supplementary material for: Effects of Combining Graphene Nanoplatelet and Phosphorous Flame Retardant as Additives on Mechanical Properties and Flame Retardancy of Epoxy Nanocomposite
Source: Polymers (Basel). 2020 Oct 14;12(10):2349. doi: 10.3390/polym12102349 (PMC7602215; doi:10.3390/polym12102349)
Supplement: Supplementary file 1 [file polymers-12-02349-s001.pdf]

Supplementary information

Effects of Combining Graphene Nanoplatelet and Phosphorous Flame Retardant as Additives on Mechanical Properties and Flame Retardancy of Epoxy Nanocomposite

Woranan Netkueakul [1,2], Beatrice Fischer [3], Christian Walder [3], Frank Nüesch [3], Marcel Rees [4], Milijana Jovic [5], Sabyasachi Gaan [5], Peter Jacob [6] and Jing Wang [1,2,*]

[1] Institute of Environmental Engineering, ETH Zurich (Swiss Federal Institute of Technology Zurich), Zurich 8093, Switzerland; woranan.netkueakul@empa.ch
[2] Laboratory for Advanced Analytical Technologies, Empa – Swiss Federal Laboratories for Materials Science and Technology, Dübendorf 8600, Switzerland
[3] Laboratory for Functional Polymers, Empa – Swiss Federal Laboratories for Materials Science and Technology, Dübendorf 8600, Switzerland; Beatrice.Fischer@empa.ch (B.F.); Christian.Walder@empa.ch (C.W.); Frank.Nueesch@empa.ch (F.N.)
[4] Laboratory for Mechanical Systems Engineering, Empa – Swiss Federal Laboratories for Materials Science and Technology, Dübendorf 8600, Switzerland; Marcel.Rees@empa.ch
[5] Additives and Chemistry Group, Advanced Fibers, Empa – Swiss Federal Laboratories for Materials Science and Technology, St. Gallen 9014, Switzerland; milijana.jovic@empa.ch (M.J.); Sabyasachi.Gaan@empa.ch (S.G.)
[6] Electronics and Reliability Center, Empa – Swiss Federal Laboratories for Materials Science and Technology, Dübendorf 8600, Switzerland; Peter.Jacob@empa.ch
* Correspondence: jing.wang@ifu.baug.ethz.ch

Received: 17 September 2020; Accepted: 10 October 2020; Published: date

Reaction between epoxy and DOPO

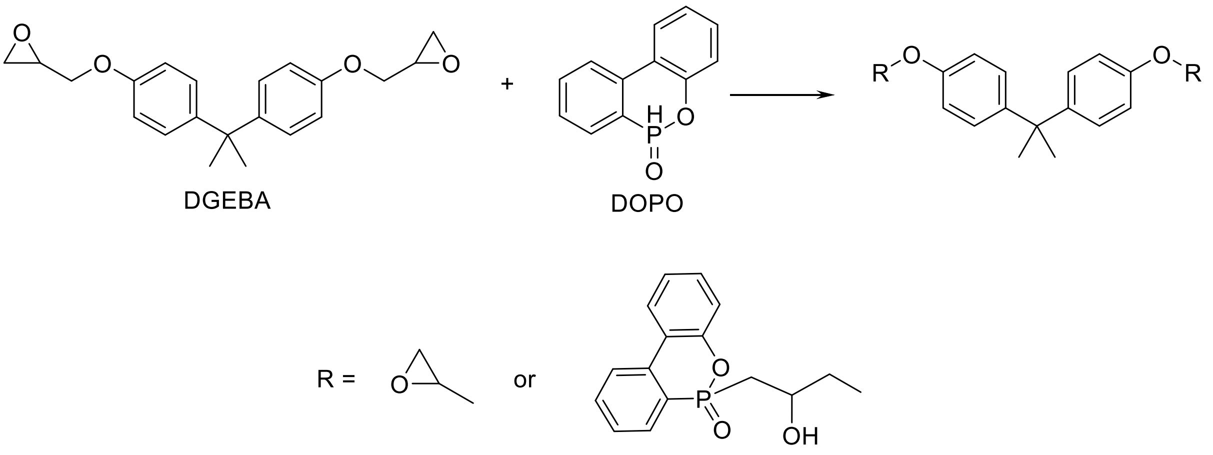

Figure S1 Reaction between epoxy resin (DGEBA) and DOPO

Calculation of epoxy/hardener ratio and epoxy equivalent weight (EEW)

The epoxy/hardener ratio was calculated according to the following equation:

$$\frac{Epoxy}{hardener} ratio = \frac{AHEW}{EEW} \times 100,$$

where amine hydrogen equivalent weight (AHEW) of Jeffamine D-230 is 59.52 g/eq;

EEW of neat epoxy= 186 g/eq (averaged value).

Therefore, the epoxy/hardener ratio is 32. For DOPO-incorporated epoxy resin, EEW value increased as the DOPO content increased due to the reaction between epoxide groups and DOPO molecules as displayed in Figure S1. The EEW can be calculated as follows:

$$EEW = \frac{Mass\ of\ epoxy\ resin + mass\ of\ DOPO}{\left(\frac{mass\ of\ epoxy\ resin}{EEW\ of\ epoxy\ resin} - \frac{mass\ of\ DOPO}{MW\ of\ DOPO}\right)}.$$

Table S1 Formulations of epoxy resin and DOPO-incorporated epoxy resin cured with polyetheramine (Jeffamine D-230)

Epoxy resin mass (g)	DOPO mass (g)	Hardener mass (g)	Phosphorous content (wt.%)
100	0	32	0
100	3	30	0.3
100	10	27	1.0
100	20	22	2.0

100	30	18	2.9

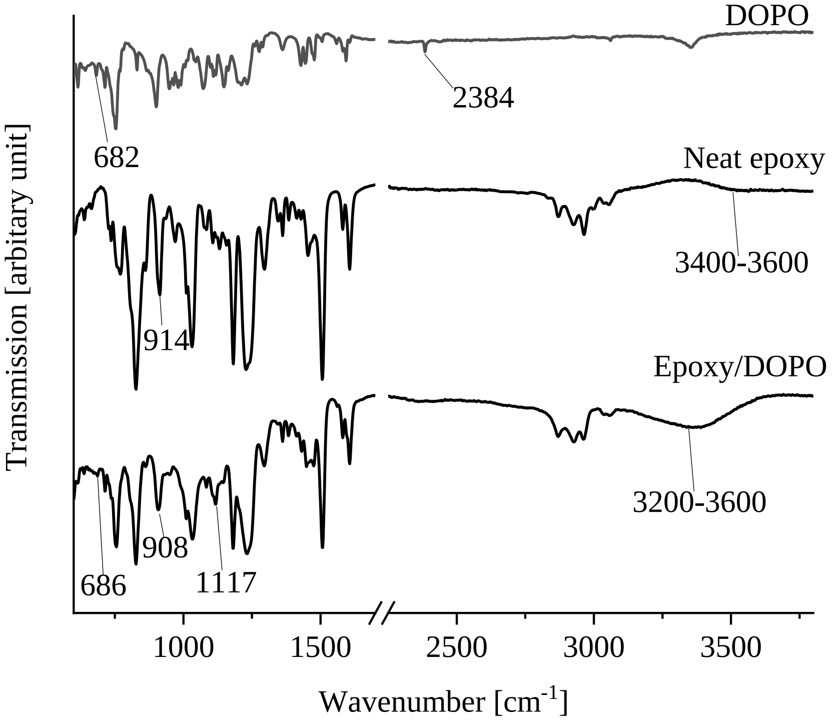

Figure S2 ATR-FTIR spectra indicating the functional groups of DOPO, neat epoxy and epoxy/DOPO mixture

Table S2 Identification of observed peaks from FTIR spectra of DOPO, neat epoxy and epoxy/DOPO mixtures

**Compound**	**Region (cm^{-1})**	**Assignment**
DOPO	3353	O-H stretching
	3060	aromatic C-H stretching
	2384	P-H stretching
	1593	aromatic C=C stretching
	1147	P=O stretching
	990	P-H bending
	753	aromatic C-H bending (ortho-substituted)
	682	P-C stretching

Neat epoxy	3200-3500	O-H stretching
	2964, 2926, 2871	C-H stretching of sp^3 CH
	1606, 1506, 1454	aromatic C=C stretching
	1581	aromatic C=C bending
	1361	sp^3 C-H bending
	1295	aromatic C-C stretching
	1182	C-C symmetrical stretching
	1031	C-O-C of ether
	914	C-O stretching of oxirane group
	827	aromatic C-H bending (para-substituted)
	770	C-H bending
EP/30DOPO	3200-3500	O-H stretching
	2962, 2927, 2869	C-H stretching of sp3 CH
	1606, 1507, 1448	aromatic C=C stretching
	1581	aromatic C=C bending
	1361	sp3 C-H bending
	1294	aromatic C-C stretching
	1181	C-C symmetrical stretching
	1117	alkoxy C-O from ring opening of epoxide group
	1033	C-O-C of ether
	908	C-O stretching of oxirane group
	826	aromatic C-H bending (para-substituted)
	755	aromatic C-H bending (ortho-substituted)
	686	P-C stretching

Electrical properties of the epoxy nanocomposites

Table S3 Electrical properties of the epoxy/GNP composites

GNP content (wt.%)	GNP content (volume fraction)	Discharge type	Measured electrical resistance (Ω)	Resistivity (Ohm-m)	Conductivity (S/m)
0.1	0.0004	sphere to sphere line spark	1.00E+12[a]	1.00E+10[b]	1.00E-10
0.5	0.0019	sphere to sphere line spark	1.00E+12[a]	1.00E+10[b]	1.00E-10
1	0.0038	sphere to sphere line spark	1.00E+12[a]	1.00E+10[b]	1.00E-10
2	0.0076	corona	7.33E+08	3.23E+06	3.10E-07
3	0.0117	corona	4.34E+07	1.91E+05	5.24E-06
4	0.0156	changing between corona and line	9.81E+05	4.32E+03	2.32E-04
5	0.0197	line	9.38E+05	4.13E+03	2.42E-04

[a] The values were measured in order of magnitude.

[b] The values were approximated using the relationship in Equation S1 and the order of magnitude of the electrode dimensions.

In order to interpret the results from the discharge test, three different phenomena can happen according to the electrical properties of each material. In case of insulating material, the hard spark can be observed between the two metal spheres. For conductive or low-resistant material, line spark originating from the metal spheres to the samples can be detected. If the sample is dissipative and has high resistance, a corona or smooth discharge can be observed between the metal spheres and the sample.

The resistance of the composites with the GNP content ranging from 0 wt.% to 1 wt.% was over the detection limit of the multimeter; therefore, the surface resistance meter (SR110, Wolfgang Warmbier GMBH & Co., Germany), which provided the measured results in orders of magnitude ranging from 10^3 Ω to 10^{12} Ω was employed. The results showed that the resistance of these

composites were in the order of 10^{12} Ω. When the GNP content increased up to 5 wt.%, the resistance decreased by seven orders of magnitude as shown in Table S3. The relationship between resistance (Ω) and resistivity (Ω-m) is presented in Equation S1.

$$\boldsymbol{R} = \boldsymbol{\rho} \times \frac{\boldsymbol{L}}{\boldsymbol{A}}$$ **Equation S1**

where R is resistance; ρ is resistivity; L is length between electrodes and A is the cross sectional area between electrodes.

The percolation threshold can be calculated according to scaling theory as shown in Equation S2, where σ_c and σ_f are the conductivity of the composite and the conductivity of the filler, respectively, Φ is the filler concentration, Φ_c is the volume fraction of filler at the percolation threshold, and t is the critical exponent that depends on the dimensionality of the GNP network. GNP conductivity used was 10^2 S/m, which was the lower bound of the conductivity reported by manufacturer (10^2 S/m when measured perpendicular to the sheet and 10^7 S/m when measured parallel to the sheet). Normally, t≈2 means GNPs form three-dimensional network [39]. This equation is only feasible when $\Phi > \Phi_c$. To calculate the volume fraction of GNP, the density of GNP, epoxy, and hardener used were 2.2 g/cm^3, 1.17 g/cm^3 and 9.47 g/cm^3, respectively.

$$\boldsymbol{\sigma_c} = \boldsymbol{\sigma_f}(\boldsymbol{\Phi} - \boldsymbol{\Phi_c})^{\boldsymbol{t}}$$ **Equation S2**

The electrical conductivity of the EP/GNP composites improved by three orders of magnitude when the GNP content increased from 1 wt.% to 2 wt.% corresponding to 0.0038 – 0.0076 volume fraction. The precipitous increase in conductivity suggested that the electrical percolation threshold should be between 1 wt.% and 2 wt.% GNP.

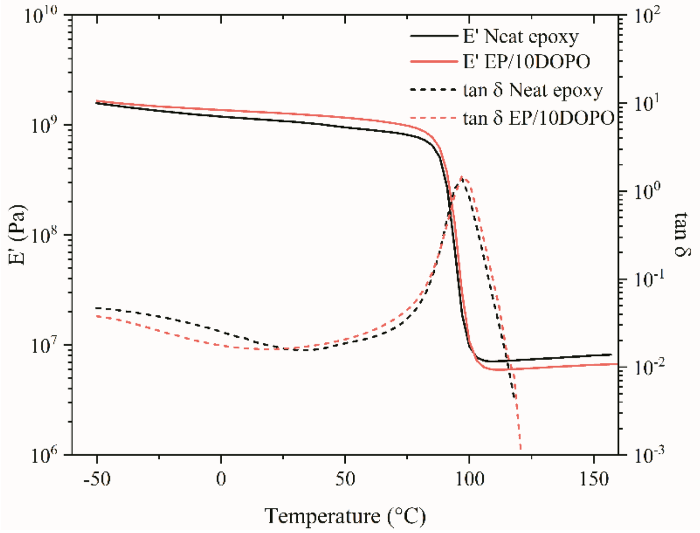

Figure S3 Storage modulus (E') and tan δ of neat epoxy resin and EP/10DOPO obtained from DMTA
